# Robust VLC Beacon Identification for Indoor Camera-Based Localization Systems †

**DOI:** 10.3390/s20092522

**Published:** 2020-04-29

**Authors:** Márk Rátosi, Gyula Simon

**Affiliations:** 1 Department of Computer Science and Systems Technology, University of Pannonia, 8200 Veszprém, Hungary; ratosi@dcs.uni-pannon.hu; 2 Alba Regia Technical Faculty, Óbuda University, 8000 Székesfehérvár, Hungary; 3 Institute for Process Systems Engineering and Sustainability, Pázmány Péter Catholic University, 1088 Budapest, Hungary

**Keywords:** indoor positioning, camera communication, visible light communication (VLC), UPSOOK, robustness, responsiveness, adaptive, autonomous vehicles

## Abstract

Several industrial indoor positioning systems utilize LEDs as beacons and cameras as sensors: The LED beacons transmit their identity, using various means of visible light communication (VLC) techniques. To avoid flickering effects, the transmission frequency is usually much higher than the sampling frequency of ordinary cameras, thus undersampling occurs. In this paper, a potential problem of undersampled protocols is highlighted: If the transmitter and receiver are not synchronized, the frequency slip between the transmitter and receiver clocks will periodically cause a burst of potential decoding errors. If the frequency slip is small (i.e., good-quality clocks are used in both the transmitter and the receiver), the time between bursts of errors is longer but at the same time the length of the bursts are also longer. An error analysis is provided as a function of protocol parameters and various error sources. Based on the results the robust-undersampled phase-shift on-off keying (UPSOOK) protocol is introduced, which guarantees the correct operation even in the presence of clock inaccuracies, as well as other error sources such as sensor noise, jitter, camera saturation, without the utilization of any error correcting codes. The properties of the proposed robust-UPSOOK protocol are demonstrated using simulations and measurements.

## 1. Introduction

For the localization of autonomous vehicles in industrial environments, novel positioning systems were proposed with LED beacons installed in known positions (e.g., on walls, ceiling), and sensors, deployed on top of the tracked vehicles. In such systems, the LED beacons transmit their identity (or equivalently, their position) using visible light communication (VLC), thus the beacon can be detected and identified by the sensor. The sensor can be either a simple photodetector (e.g., a photodiode) or a more complex two-dimensional image sensor (e.g., a camera). Photodetectors offer inexpensive solution with possibly high data rate. The sensed quantity in this case is the received signal strength (RSS), which can be used for ranging, with moderate accuracy. Based on the estimated distances, the localization is performed using some kind of trilateration [1,2].

**Figure 1:**
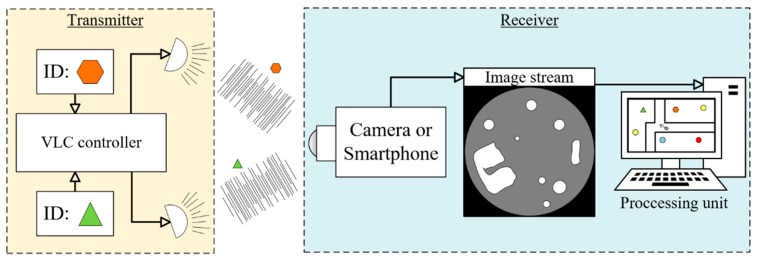
Localization system utilizing LED beacons deployed in known locations, and a moving camera installed on top of the autonomous vehicle.

Image sensors, on the other hand, are more expensive, offer much lower data rate, but offer rich sensing possibilities, e.g., the image position, shape, or angle can be measured on the sensor image, in addition to the RSS [3,4,5,6,7]. Depending on the measured quantities, various positioning techniques can be used to determine the camera position. The localization accuracy is possibly much better than in case of the RSS-based systems. An example is shown in Figure 1, where angle of arrival (AoA) or angle difference of arrival (ADoA) methods are utilized [3,4,5,6].

The main challenge in such camera communication methods is that the blinking of the LEDs should be invisible for the human eye (flicker-free operation), while at the same time, ordinary cameras should be able to detect and decode the transmitted code. These protocols must operate over long distances (several tens of meters), but the required communication rate is low (a few bytes/s is satisfying), as opposed to protocols designed for short distance and high data rate. Several undersampled communication schemes have been proposed to satisfy such requirements [8,9,10,11,12,13].

A rarely emphasized requirement, in connection with VLC systems, is responsiveness. In real-time positioning systems (e.g., autonomous forklifts in industrial applications), beacons should be detected and identified shortly after they become visible (e.g., after entering a room through a door), preferably within a guaranteed response time. In this paper, protocols will be analyzed with this requirement in mind, and a new protocol will be proposed, which provides guaranteed response time in the presence of inaccuracies in the physical equipment.

First, we will highlight a possible weakness of current undersampled on-off-keying (OOK) protocols in applications where guaranteed response time is required with high reliability. We will demonstrate that these protocols are very likely to fail periodically, and also analyze the reason behind this phenomenon: The failure is primarily due to the combined effect of the integral sampling of the camera and the slight frequency slip present between the transmitter’s and the receiver’s clocks. The presence of noise, jitter, and sensor saturation all increase the probability of the failure. We will use the Undersampled Phase Shift ON-OFF Keying (UPSOOK) protocol [9] for demonstration, since its elements will be utilized later in the proposed protocol. Notice that the discussed effect is not specific to UPSOOK, but is can be observed in other undersampled protocols as well [14].

In practical applications, where the transmitters and receivers are not synchronized, the frequency slip is always present. We will show that the larger the frequency slip, the more frequent the failure, while on the other hand, the smaller the slip, the longer the failure lasts. This second phenomenon is especially dangerous in systems where responsiveness is a key requirement. The properties of the error bursts will be analyzed as a function of protocol parameters and the properties of the error sources.

Using the lectures learned during the analysis, a robust protocol will be introduced, based on the preliminary results of [15]. The robust UPSOOK (RUPSOOK) protocol provides reliable connection, and thus guarantees response time in the presence of such common errors as noise, jitter, saturation, and frequency slip. The performance properties will be demonstrated through simulations and real measurements. The pros and cons of the proposed protocol are the following:

+ The protocol is insensitive to various error sources, providing guaranteed response time in a wide range of operating conditions;
+ The protocol does not utilize special symbols for header, thus higher reliability can be achieved for longer distances,
– The data rate is reduced by a factor of approximately 3.5, with respect to UPSOOK.

This paper builds on results presented in [15], and provides the following new contributions:

- The adaptive version of RUPSOOK is presented, which provides robust operation when the distance between the beacon and sensor is changing and thus the detected beacon intensity is changing in time;
- The error analysis is extended, now containing effects of noise, jitter, frequency error, saturation, and suboptimal thresholding;
- The evaluation is extended, containing simulations and real measurements.

The rest of the paper is structured as follows: In Section 2, related work will be reviewed concerning VLC in localization systems, with special emphasis on undersampled protocols, in particular UPSOOK. In Section 3, we will analyze errors in UPSOOK (and in general, undersampled OOK protocols). In Section 4, the RUPSOOK protocol will be introduced. The performance evaluation in Section 5 contains a design example and several measurements. Section 6 concludes the paper.

## 2. Related Work

### 2.1. VLC Beaconing in Camera-Based Localization Systems

The general architecture of localization systems using VLC and camera is shown in Figure 1. The beacons are LEDs, which transmit their identity using VLC. Usually, several such beacons are deployed in known positions. The moving camera (which can be, for example, an embedded camera of a smart phone or an industrial camera deployed on top of an autonomous vehicle) creates an image stream, which is used to detect and identify beacons. Based on the detected beacon positions on the image, and the known locations of the beacons, the processing unit estimates the location of the camera. 

Luxapose [16] uses the rolling shutter phenomenon to identify beacons on the image. This method is applicable if the beacons are close to the sensor, so that the image of the beacon is large enough for decoding; the maximum distance between the camera and the beacon is only a few meters. The system in [1] utilizes an image sensor to sense the beacons and an accelerometer to determine the tilting angle of the camera. From this information, the position of the camera is determined. The Lookup system [4] utilized long-distance beaconing with an up-facing fisheye camera, with robust sensor fusion. With the auxiliary accelerometer and magnetometer (to estimate inclination), the system is able to use as few as 1 or 2 beacons for localization, while more beacons allow fault tolerant location estimation. In [5], the inclination of the camera is estimated without using auxiliary sensors if at least four beacons are visible.

The camera measurements are transformed to position estimates using various position estimation methods, e.g., exhaustive search [5], least squares [1,5], consensus-functions [4], or Perspective-n-Point algorithms [6].

### 2.2. Undersampled Protocols for Camera Communication

To provide flicker-free operation for ordinary cameras (where the sampling rate can be as low as 30 Hz), special undersampled coding schemes must be used. In the literature, several undersampled modulation schemes have been proposed. Undersampled Frequency Shift ON-OFF Keying (UFSOOK) [8] used frequency modulation with two specially designed frequencies for data coding, and a third frequency for header. The header frequency is high, so the camera senses it as half-intensity signal. Each bit is decoded using two consecutive samples: The coding frequencies are chosen that in case of bit 1 the two samples are identical, while in the case of bit 0, they are different. UPSOOK [9] uses phase modulation; here, one bit is decoded using one sample (plus the header). In UPSOOK, only one frequency is used for data coding and the bits are coded with 0 or 180° of phase shift in the transmitted signal. The detailed operation will be described in Section 2.3. The phase modulation was combined with amplitude modulation in [10], thus one transmitted symbol can decode multiple bits, providing higher bitrate. The amplitude modulation can be replaced with high-frequency pulse-width modulation, allowing simpler transmitter hardware [11].

These techniques provide low to moderate bitrates in the range of 10 bps–100 bps with an ordinary 30 FPS camera within a few meters. Protocols with higher bandwidth, using various color coding and parallel channels, were also proposed (e.g., [12,17]), but for the target application field, these are not applicable due to their limited communication range.

The longest communication range can be achieved by the simple and robust OOK methods of UFSOOK and UPSOOK. Since our proposed protocol is related to UPSOOK, this protocol will be reviewed in detail in the following section.

### 2.3. The UPSOOK Protocol

**Figure 2:**
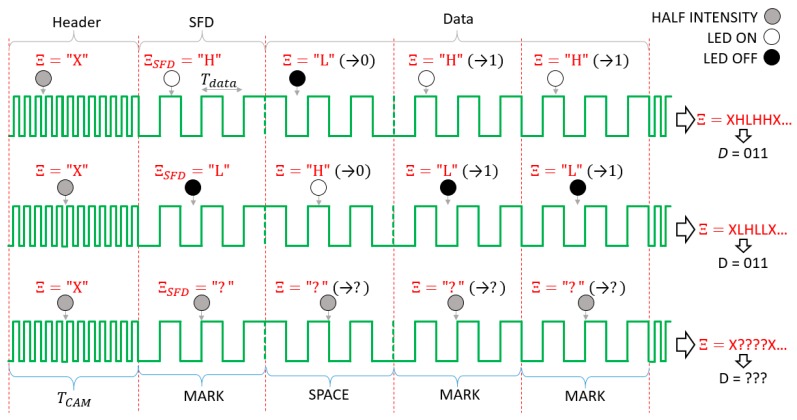
The operation of the undersampled phase-shift on-off keying (UPSOOK) protocol. The sent packet contains header (X), start frame delimiter (SFD = 1), and three data bits of 011. Due to the phase uncertainty, two possible received sample sequence can be produced (**first** and **second** rows), where the ambiguity can be resolved using the SFD. When sampling is done near the edges, the outcome of the decoding is uncertain (**third** row).

The operation of the UPSOOK protocol is illustrated in Figure 2. In the protocol, three symbols are utilized. Both MARK and SPACE symbols are square wave signals with a duty cycle of 50% and a frequency of

(1) $$f_{data}=nf_{CAM},$$

where n is integer and $f_{CAM}$ is the camera sampling frequency (in the illustration of Figure 2 the parameter n is 3). Equation (1) can be rewritten to represent the relationship between the signal period $T_{data}$ and the sampling interval $T_{CAM}$:

(2) $$nT_{data}=T_{CAM}.$$

The phases of MARK and SPACE symbols differ by 180°, as shown in Figure 2.

The third type of symbol is utilized to identify the header: It is also a square wave signal with duty cycle of 50% but with much higher frequency $f_{header}$. The signal frequencies are determined so that

(3) $$f_{data}\ll \frac{1}{S}$$

and

(4) $$f_{header}\gg \frac{1}{S}.$$

where *S* is the camera exposure time. When (3) holds, then the camera senses that the signal level is either high (H) or low (L), while with (4), the sensed signal level X has approximately half intensity. Notice that this is true for the ideal case, but in practice, it was found that the level of X approaches the level of H, as the communication distance increases [9]. This makes decoding more difficult and limits the maximum communication distance of UPSOOK.

The transmitted packet contains a header symbol, followed by the start frame delimiter (SFD), which is always a MARK symbol, and then one symbol for each data bit. Data bits are simply coded as follows: 1: MARK, 0: SPACE. See the example of Figure 2 for data bit sequence of 011.

Each symbol has length of $T_{CAM}$, thus exactly one sample is taken from each symbol. The received symbols are denoted by Ξ. Since the transmitter and receiver are asynchronous, sampling can happen at any phase of the signal. The phase uncertainty does not affect the X symbol, due to (4), but it does affect the SFD and data symbols. Figure 2 illustrates the two possible outcomes: In the first case, the received SFD is $\Xi_{SFD}=H$ and the received data symbols are $\Xi_{DATA}=LHH$ (upper row of Figure 2), while in the second case, $\Xi_{SFD}=L$ and the data symbols are $\Xi_{DATA}=HLL$ (middle row of Figure 2). Using the SFD, the ambiguity of the data bit $\Xi_{D}$ is resolved in the following way:

(5) $$D=\{\begin{array}{cc} 1 & \mathrm{if}\;\Xi_{SFD}=\Xi_{D}\;\\ 0 & \mathrm{if}\;\Xi_{SFD}\neq {\;\Xi }_{D} \end{array}.$$

where D is the bit value corresponding to symbol value $\Xi_{D}$. In the example of Figure 2, both cases result in the correct bit sequence of 011.

The camera detects light intensity $I_{s}$, which is a value between 0 and $2^{B}-1$, where B is the depth of the camera sensor (in bits). From $I_{s}$ the detected symbols are generated by thresholding, as follows:

(6) $$\Xi =\{\begin{array}{cc} H & \mathrm{if}\;I_{s}\geq Q\;\\ 0 & \mathrm{if}\;I_{s}<Q \end{array},$$

where Q is the decision threshold.

Notice that the decoding works only if the sampling is close to the ideal circumstances shown in the first two lines of Figure 2. If samples are taken around the signal edges, as shown in the third line of Figure 2, erroneous detections can happen. The error sources and their effects will be analyzed in the next section.

## 3. Error Analysis

### 3.1. Camera Model

**Figure 3:**
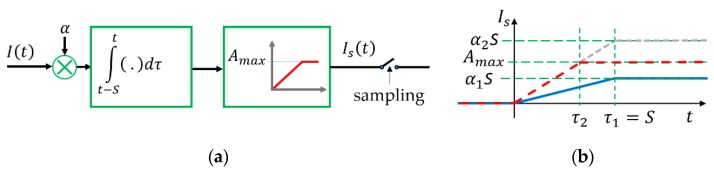
Camera operation model. (**a**) Mathematical model, (**b**) step response of the camera: Blue, without saturation; red dashed, with saturation.

To allow the analysis of the effects of various error sources on undersampling protocols, a mathematical model of the camera operation is utilized, illustrated in Figure 3a.

The incoming light intensity is denoted by I(t), which is multiplied by α, representing the cumulative effect of several camera properties (e.g., aperture, gain). The camera sensor integrates the incoming light during aperture time S. The highest value the sensor can represent is $A_{max}$, corresponding to incoming light intensity $I_{max}$: Light intensity values higher than $I_{max}$ cause saturation in the sensor and produce output value $A_{max}$. Thus, the mathematical model of the camera operation is the following:

(7) $$I_{s}\left(t\right)=\min\left(\alpha \overset{t}{\underset{t-S}{\int }}I\left(\tau \right)d\tau ,\;A_{max}\;\right).$$

An important implication of (7) is the following: The detected light intensity is not the sampled incoming light intensity, but rather its integral over a time period of S. Thus, if the input signal is a step function then, according to (7), $I_{s}\left(t\right)$ will be a ramp signal with slope of α. If there is no saturation, then the length of the rising edge is $\tau_{1}=S$, as shown in Figure 3b (blue line). If the sensor is saturated, then the length of the ramp will be shorter, as shown in Figure 3b by the dashed red line. In this case, the length of the rising edge is $\tau_{2}=A_{max}/\alpha_{2}\;$.

### 3.2. Error Sources

The first two rows of Figure 2 show an ideal situation when the sampling is done near the middle of the light and dark pulses. However, this cannot be guaranteed; if the transmitter and receiver are not synchronized, the sampling may be performed near the edges of the blinking signal, as shown in the third row of Figure 2.

The sampling around the edges may be problematic, since the signal’s transition time is not instantaneous, as was shown in Section 3.1. The sample taken during the signal’s transition may be detected incorrectly. The outcome of the detection depends on the decision threshold, noise, jitter, frequency error, and the possible saturation of the signal. The possible error sources and their effect will be discussed in detail.

#### 3.2.1. Threshold

**Figure 4:**
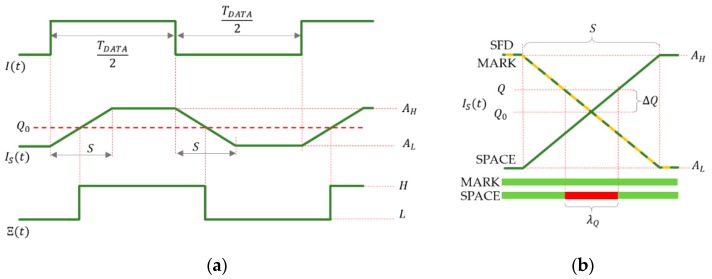
Decoding of symbols using thresholding. (**a**) Using ideal threshold $Q_{0}=\left(A_{H}-A_{L}\right)/2$. (**b**) The effect of non-ideal threshold: Samples taken in the unsafe interval (**red**) provide incorrect symbols, while samples in the safe regions (green) provide correct symbols.

If I(t) is a rectangular signal with duty cycle of 50% and there is no saturation, then, according to (5), $I_{s}\left(t\right)$ is a symmetric trapezoid signal with rising and falling times equal to S, as shown in Figure 4a. Notice that the symbol coded by signal I(t) can be reconstructed from $I_{S}\left(t\right)$ by using the ideal threshold

(8) $$Q_{0}=\frac{A_{H}-A_{L}}{2}.$$

Using $Q_{0}$, the time function of the received symbol is the exact replica of that of the transmitted symbol, delayed by S/2, as shown in Figure 4a.

The illustration of Figure 4b shows multiple signal segments of $I_{s}\left(t\right)$ around signal changes, so that the shown signal segments are $kT_{CAM}$ apart, where k is integer. If (1) holds, then all rising edges and all falling edges are precisely aligned, as shown in Figure 4b. The horizontal axis represents possible sampling instants and at the same time successive sampling instants $kT_{CAM}$ time later. Using the figure, the values of the samples can be determined, given the sampling instant and the threshold.

As an example, Figure 4b shows an SFD (around a falling edge, shown by orange line), a MARK symbol (around a falling edge, dashed green line), and a SPACE symbol (around a rising edge, solid green line). The ideal threshold $Q_{0}$, and the real threshold Q are also shown.

Let us suppose that the data symbol to be decoded is SPACE. In this case, the samples, taken from the SFD and from the data symbol, should be different for correct detection. This is true for time instants both on the left-hand side and the right-hand side of Figure 4b. However, if samples are collected in the unsafe time interval, shown by red in Figure 4b, then both samples are considered L, resulting in incorrect detection. In Figure 4b a falling SFD edge and a rising data edge are shown; notice that similar unsafe intervals exist on the opposite edges, too.

If the detected signal amplitude is $A_{L}$ and $A_{H}$ for symbols L and H, respectively, and the difference between the actual and ideal thresholds is $\Delta Q=Q-Q_{0}\;$, then in worst case, the width of the unsafe interval can be computed, using similar triangles of Figure 4b, as follows:

(9) $$\lambda_{Q}=\frac{2\left|\Delta Q\right|}{A_{H}-A_{L}}S.$$

If the data symbol is MARK and no other disturbances are present, then the sample of the SFD and the sample of the data symbol will be the same (see the yellow and dashed green lines in Figure 4b); in this case, the value of Q has no effect on the decoding, i.e., for MARK symbols there is no unsafe interval. Since in a message, both MARK and SPACE symbols may be present, in the worst case, (9) provides the width of the unsafe interval.

#### 3.2.2. Noise

**Figure 5:**
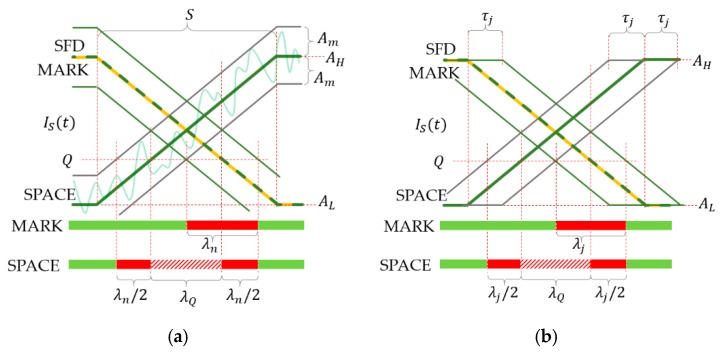
The effect of noise and jitter on symbol decoding. Samples taken in the red unsafe intervals may provide incorrectly decoded symbols, while samples in the safe regions (green) provide correct detections. (**a**) Effect of noise, (**b**) effect of jitter.

The sensed signal $I_{s}\left(t\right)$ may contain additive noise, due to possible external disturbances and camera sensor noise. The effect of noise is illustrated in Figure 5a, where the maximum noise amplitude is denoted by $A_{n}$. For MARK symbols, the width of unsafe interval, shown by a red line (for the case of no saturation), using notations of Figure 5a, is the following:

(10) $$\lambda_{n}=\frac{2A_{n}}{A_{H}-A_{L}}S.$$

According to the results of Section 3.2.1, for SPACE symbols, there may exist an unsafe interval, due to inaccurate thresholding, denoted by a striped red line in Figure 5a. The additive noise widens this interval on both sides, as shown by red intervals in the figure. The width of the red intervals on both sides are equal to $\lambda_{n}/2$, where $\lambda_{n}$ is defined in (10). Thus, the total increase of the unsafe interval, due to noise, equals to $\lambda_{n}$ for both MARK and SPACE symbols.

#### 3.2.3. Jitter

Both the transmitter and the camera may have jitter. The effect of the cumulative jitter is illustrated in Figure 5b. Let us use the SFD as reference; in the presence of jitter, the edges of SPACE and MARK symbols may arrive earlier or later than in the ideal case, thus the sample taken in the unsafe interval may or may not be correct. If the maximum jitter is $\tau_{j}$, then the width of the resulting unsafe interval for MARK symbols is the following:

(11) $$\lambda_{j}=2\tau_{j}.$$

For SPACE symbols, very similarly to the case of noise, jitters enlarge the unsafe interval of inaccurate thresholding. The total growth of the unsafe interval is equal to $\lambda_{j}$ of (11), as shown in Figure 5b. Thus, for both MARK and SPACE symbols the effect of jitter is characterized by (11).

#### 3.2.4. Frequency Error

**Figure 6:**
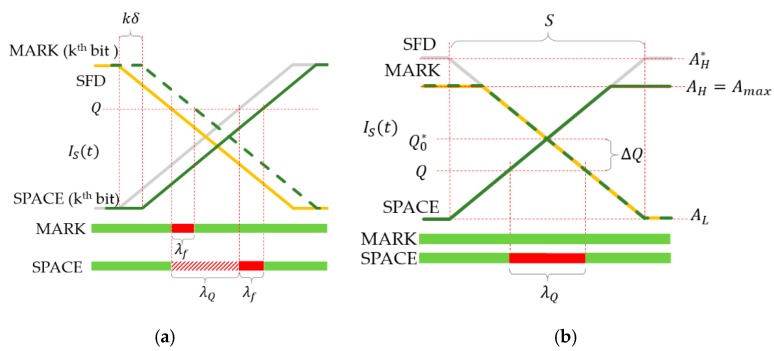
Effect of frequency error and saturation. SFD: Orange, SPACE: Solid green, MARK: Dashed green. Samples taken in the red unsafe intervals provide incorrectly decoded symbols, while samples in the safe regions (green) provide correct detections. (**a**) The effect of frequency error on symbol decoding. Grey line shows the SPACE symbol with no frequency error. (**b**) The effect of saturation. Gray lines show the ideal signals without saturation.

Ideally, the camera’s sampling interval $T_{CAM}$ is an integer multiple of the blinking period length $T_{data}$, according to (2). The validity of (2) can be ensured if the transmitter and receiver are synchronized. In practical cases, where no time synchronization is applied, there always is a small frequency error between the transmitter and the camera, i.e., instead of (2), the following holds:

(12) $$T_{CAM}=nT_{data}+\delta ,$$

where δ can be considered as the error of the camera’s sampling interval when the transmission frequency is accurate. If δ is not zero, the phase of the sampling instants is shifted at each sample, the effect of which is shown in Figure 6a for the *k*th data bit. Notice that during decoding each sample is compared to the SFD, according to (5). Thus, the reference is the SFD sample, and each of the successive sampling times of the data bits are shifted by δ, the total delay being kδ at the *k*th data bit, as shown in Figure 6a. Thus, compared to the reference SFD, the first bit is sampled δ later than ideal; the delay of the second bit is 2δ, etc., generally the kth bit is sampled kδ later. Thus, the unsafe interval for the kth bit, due to frequency error, for both the SPACE and MARK symbols is the following:

(13) $$\lambda_{f}=k\delta .$$

#### 3.2.5. Saturation

Let the maximum incoming light intensity, which does not cause saturation, be $I_{max}$, i.e., according to (7), the following equality holds:

(14) $$A_{max}=\alpha SI_{max}$$

Let the input light intensity be $I=qI_{max}$, where the overload factor q>1. In this case $A_{H}=A_{max}$, and we assume that $A_{L}<A_{max}$. However, without saturation, the value of $A_{H}$, according to (7), would be

(15) $$A_{H}^{*}=qA_{max}.$$

In this case, instead of (8), the ideal threshold value is given by

(16) $$Q_{0}^{*}=\frac{A_{H}^{*}-A_{L}}{2}=\frac{qA_{max}-A_{L}}{2}.$$

The ideal threshold can be realized only if $Q_{0}^{*}<A_{max}$. Following from (16), the constraint for the overshoot factor q is the following:

(17) $$q<2-\frac{A_{L}}{A_{max}}.$$

In the common case when $A_{L}≅0$, constraint (17) leads to q<2. If (17) is satisfied, it is theoretically possible to apply the ideal threshold $Q_{0}^{*}$. However, for this the overshoot factor, q must be known, which in most practical cases is not feasible (e.g., when an autonomous vehicle is moving it is hard to estimate the exact overshoot factor, even if the location is known). If (17) is violated (i.e., very high saturation), then there is no way to apply $Q_{0}^{*}$ to the detector; in this case, ΔQ will always be non-zero. 

When saturation is present, the unsafe interval width estimate (9), due to inexact thresholding, is replaced by the following:

(18) $$\lambda_{Q}^{*}=\frac{2\left|Q-Q^{*}\right|}{A_{H}^{*}-A_{L}}S.$$

The effect of noise can be estimated with the following equation, replacing (10):

(19) $$\lambda_{n}^{*}=\frac{2A_{n}}{A_{H}^{*}-A_{L}}S.$$

The effects of jitter and frequency error are not modified by saturation.

### 3.3. Performance Properties

**Figure 7:**
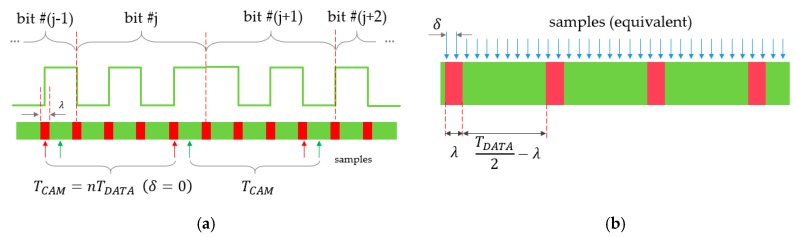
(**a**) Unsafe intervals with width of λ, as a cummulative results of various error sources. Sampling is made with δ=0. Red arrows: All samples are taken in unsafe intervals; green arrows: All samples are taken in safe intervals. (**b**) The equivalent sampling of the signal with equivalent time interval of δ>0. Samples are taken from safe and unsafe intervals as well. The unsafe and safe intervals are red and green, respectively.

In the worst case, the cumulative effect of the above error sources can be represented as a combined unsafe interval with width of λ, around the edges of the transmitted signal, as shown in Figure 7. In the worst case, the width of the combined unsafe interval is the following for the case of non-saturated camera:

(20) $$\lambda =\lambda_{Q}+\lambda_{n}+\lambda_{j}+\lambda_{f},$$

while the worst-case saturated case is described by the following equation.

(21) $$\lambda =\lambda_{Q}^{*}+\lambda_{n}^{*}+\lambda_{j}+\lambda_{f},$$

When the frequency synchronization between the transmitter and the camera is perfect, according to (2), the sampling is always performed at the same phase of the transmitted signal, thus the sampling is either always good (green arrows of Figure 7a), or always possibly bad (red arrows of Figure 7a). In practical cases, there always is a small frequency error between the transmitter and the camera, i.e., (12) holds. If δ is not zero, the phase of the sampling instants is shifted at each sample, as shown in Figure 7b. When δ>0, the equivalent samples are taken from left to right, while in the case of δ<0, from right to left. In Figure 7b the safe and unsafe intervals are shown by green and red colors, respectively, and the equivalent sampling instants are denoted by blue arrows. The interpretation of the figure is the following: If a sample is taken at a particular phase of the signal (e.g., the leftmost blue arrow of Figure 7b), then the next sample will be taken δ time to the right (second blue arrow in the middle of the unsafe interval in Figure 7b), the next sample is taken further δ time to the right (third arrow at the beginning of the safe interval in Figure 7b), etc. The samples traverse along the alternating safe and unsafe intervals; thus, the sampling is performed in the safe interval for a while, then in the unsafe interval, then again in the safe interval, etc.

The number of samples in the unsafe region is

(22) $$N_{unsafe}=\frac{\lambda }{\delta },$$

while in the safe region the number of samples is

(23) $$N_{safe}=\frac{T_{data}}{2\delta }-\frac{\lambda }{\delta }.$$

Thus, the transmission is potentially broken periodically with period

(24) $$T_{P}=T_{CAM}\left(N_{unsafe}+N_{safe}\right)=T_{CAM}\frac{T_{data}}{2\delta }$$

and the length of the potentially bad interval is

(25) $$T_{unsafe}=T_{CAM}N_{unsafe}.$$

While the sampling is done in the unsafe interval, the protocol is likely to provide incorrect detections, thus the protocol may not be responsive for time interval $T_{unsafe}$. Thus, the responsiveness in worst case is characterized by $T_{unsafe}$.

### 3.4. Illustration

The responsiveness of the UPSOOK protocol was tested in laboratory settings in ideal circumstances. In the test setup, 9-bit packets were transmitted repeatedly. Altogether, 45,000 bits (5000 packets) were sent, using $T_{data}=\frac{n}{30\;s}$ and $T_{CAM}=\frac{1}{30\;s}+\delta$, where n=4, δ=0.2 μs and S=250 μs. The detected signal amplitude was between $A_{L}=7$ and $A_{H}=109$. The standard deviation of the measurement noise was $\sigma_{noise}=0.6\;\mathrm{LSB}$.

**Figure 8:**
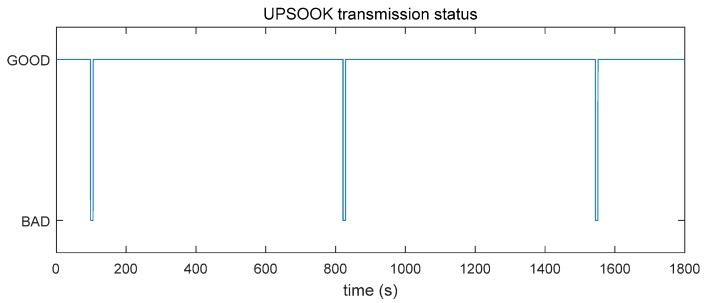
Measured transmission status of the UPSOOK protocol, with S=250 μs, $A_{L}=7$, $A_{H}=109$, Q=50, and  δ=0.2 μs.

To illustrate the behavior of UPSOOK, a test was run with various values of Q. The transmission status of the protocol for Q=50 is shown in Figure 8. As shown in the record, the protocol fails periodically, where $T_{P}≅12\;\min$, and the width of the unsafe intervals was $T_{unsafe}≅7\;s$. During these unsafe intervals, the system is not responsive: In the worst case, the detection of a beacon is delayed by 7 s.

Table 1 contains measured and theoretical values for $T_{P}$ and $T_{unsafe}$, for Q=20, 50 and 58. The measured and theoretical values show good correspondence, validating the theoretical results.

## 4. Robust UPSOOK

Now, a robust version of UPSOOK will be introduced. First, the operation of the protocol will be described, followed by the robust design of the protocol parameters. Then, the adaptive thresholding mechanism will be introduced, and finally, a design example will be presented.

### 4.1. RUPSOOK Protocol

**Figure 9:**
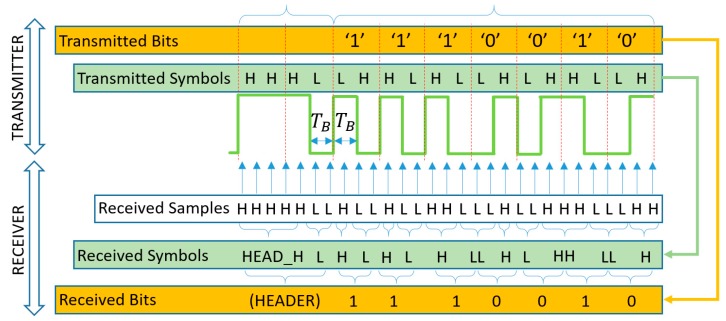
The operation of the robust UPSOOK protocol.

The protocol uses only two symbols, which are single ON (H) and OFF (L) states of the transmitter, each symbol having a width of $T_{B}$, as shown in Figure 9. First, the data bits to be transmitted are Manchester-coded, i.e., each bit is represented by two symbols as follows:

(26) $$\begin{array}{cc} 0: & \mathrm{LH}\\ 1: & \mathrm{HL} \end{array}$$

The Manchester coded bits can be considered as phase shift keying signals, similarly to UPSOOK’s MARK and SPACE symbols. The main difference is that, according to (2), each UPSOOK symbol contains n>1 periods, while here, only one period is utilized, as shown in Figure 9.

At the beginning of the packet, the header is transmitted, which contains $N_{HEAD}=4$ symbols of HHHL. Due to the Manchester coding, the three consecutive H symbols can never be present in the data part, thus the header can be separated from the data (see details of decoding later). The data segment, following the header, contains B bits, Manchester encoded.

The packet contains M symbols, where

(27) $$M=N_{HEAD}+2B$$

**Figure 10:**
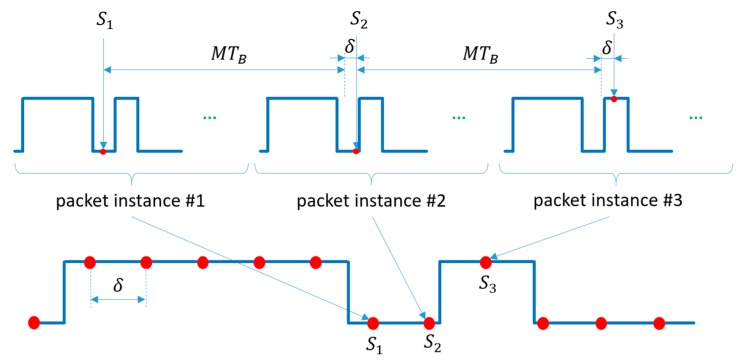
The sampling at the receiver side of robust UPSOOK protocol, and its equivalent representation. The equivalent sampling interval is δ.

The transmitter repeats the packet of M symbols continuously. The receiver is configured to have an intentional frequency shift between the transmitter and receiver; the sampling interval $T_{CAM}$ of the receiver is set so that $T_{CAM}$ is slightly longer than $T_{B}$:

(28) $$T_{CAM}=T_{B}\left(M+c\right),$$

where 0<c<1. With this setting, the continuously transmitted packet will be sampled so that the phase of the samples changes continuously, providing equivalent sampling intervals of

(29) $$\delta =cT_{B}$$

between consecutive samples, as illustrated in Figure 10. If δ were zero, each instance of the packet would be sampled at the same phase. Since δ>0, instance k+1 is sampled at a different phase, virtually δ time later than instance k. The equivalent sampling of the packet is shown in the lower part of Figure 10.

### 4.2. Robust Parameter Design

The value of c should be chosen so that the following three requirements are fulfilled:

**R1**: From each symbol, one or two samples are taken;
**R2**: From two consecutive symbols, three or four samples are taken;
**R3**: From three consecutive symbols, at least five samples are taken.

The above requirements allow the decoding of the symbols from the received samples, as shown in the lower part of Figure 8. First, the number of the same consecutive samples is determined. In Figure 8, the illustration shows five L, two H, one L, etc., samples in row *Received Samples*. Using R1–R3, the symbols are decoded as follows:

- One or two of samples Y is converted to a symbol Y, where Y is L or H.
- Three or four of samples Y is converted to symbols YY, where Y is L or H.
- At least five consecutive samples of H are converted to HEAD_H.

The result of symbol decoding is illustrated in Figure 8, row *Received Symbols*. The received symbols are then converted to bits, using the following rules:

- A HEAD_H, followed by L, is the HEADER.
- After the HEADER, B symbol pairs are decoded using (26).

The decoded bits are shown in Figure 8 in row *Received Bits*.

The value of constant c must be determined so that the protocol be tolerant towards the error sources. Most of the error sources, described in Section 3.2, are relevant for RUPSOOK as well: The effects of thresholding, noise, jitter, and saturation on the sampling are the same. The frequency error, which was characterized by the slip parameter δ, is now a design parameter. Thus, the width of the unsafe regions is now estimated by (20) or (21), with $\lambda_{f}=0$.

**Figure 11:**
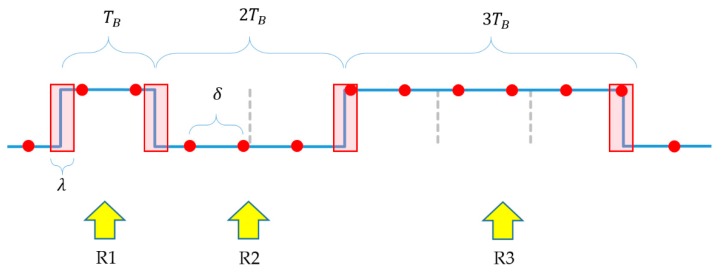
Unsafe regions (red rectangles) in a transmitted signal, showing the equivalent sampling of the signal (red dots). The example also illustrates requirements R1, R2, and R3.

In the protocol one, two, or three of the same symbols can follow each other (one or two in the data segment, three in the header). Since sampling around the edges is unsafe, again safe and unsafe regions are observed, as shown in Figure 11: The symbols’ width is $T_{B}$, and the unsafe regions’ width is  λ. 

Notice that the unsafe regions may virtually increase or decrease the width of a symbol in the worst case by λ. For example, in Figure 11, if the sample taken in the third unsafe interval (middle of the figure) is L, then the sensed length of two symbols L becomes longer and at the same time the length of the three symbols H seems shorter. If the sample in the unsafe interval is H, then the effect will be the opposite. 

Taking this effect into consideration, R1 can be expressed as follows (for illustration see the first symbol H in Figure 11):

(30) $$T_{B}-\lambda >\delta$$

(31) $$T_{B}+\lambda <2\delta$$

Similarly, for R2 (see the two consecutive symbols L in Figure 11):

(32) $$2T_{B}-\lambda >3\delta$$

(33) $$2T_{B}+\lambda <4\delta$$

Finally, R3 results in the following constraint (see the three consecutive symbols H in Figure 11):

(34) $$3T_{B}-\lambda >5\delta$$

From (30), (32) and (34) it follows:

(35) $$\delta <\min\left(T_{B}-\lambda ,\;\frac{2}{3}T_{B}-\frac{1}{3}\lambda ,\;\frac{3}{5}T_{B}-\frac{1}{5}\lambda \right)$$

Since, in a meaningful case $\lambda <T_{B}/2$, (35) can be simplified to

(36) $$\delta <\frac{3}{5}T_{B}-\frac{1}{5}\lambda .$$

From (31) and (33), the following constraint follows:

(37) $$\delta >\max\left(\frac{T_{B}}{2}+\frac{\lambda }{2},\frac{T_{B}}{2}+\frac{\lambda }{4}\right)=\frac{T_{B}}{2}+\frac{\lambda }{2}.$$

Let us use the following notation:

(38) $$\lambda =aT_{B}.$$

Using (29) and (36)–(38), the possible region for constant c is the following:

(39) $$\frac{1}{2}\left(1+a\right)<c<\frac{3}{5}\left(1-\frac{a}{3}\right).$$

The solution exists if

(40) $$a<\frac{1}{7}.$$

From (38) and (40), it follows that the protocol can operate only if the unsafe interval λ is smaller than $T_{B}/7$. From (39), a region is given for possible values of c, where the width of the region is

(41) $$c_{max}-c_{min}=\frac{1}{10}-\frac{7}{10}a$$

The value of c should be chosen as the middle of the interval:

(42) $$c_{opt}=0.55+0.15a$$

### 4.3. Adaptive Thresholding

In case the amplitudes $A_{L}$ and $A_{H}$ change in time, the ideal value of $Q_{0}$ also changes. We propose the following adaptive estimator for $Q_{0}$. Let the sampled intensity value at time instant k be $I_{S}\left(k\right)$. Let us use a time window with length W. The minimum and maximum amplitudes are estimated as follows:

(43) $$I_{MAX}\left(k\right)=\max\left(I_{S}\left(k-W+1\right),\;I_{S}\left(k-W+2\right),\ldots ,I_{S}\left(k\right)\right),$$

(44) $$I_{MIN}\left(k\right)=\min\left(I_{S}\left(k-W+1\right),\;I_{S}\left(k-W+2\right),\ldots ,I_{S}\left(k\right)\right),$$

and the estimate of $Q_{0}$ is the following:

(45) $$Q_{0}\left(k\right)=\frac{I_{MAX}\left(k\right)+I_{MIN}\left(k\right)}{2}.$$

The window length W must be chosen so that the window contains at least one sample from a symbol L and at least one sample from a symbol H, at any part of the signal, even in the header. This requirement is satisfied if W>2c≈8.

**Figure 12:**
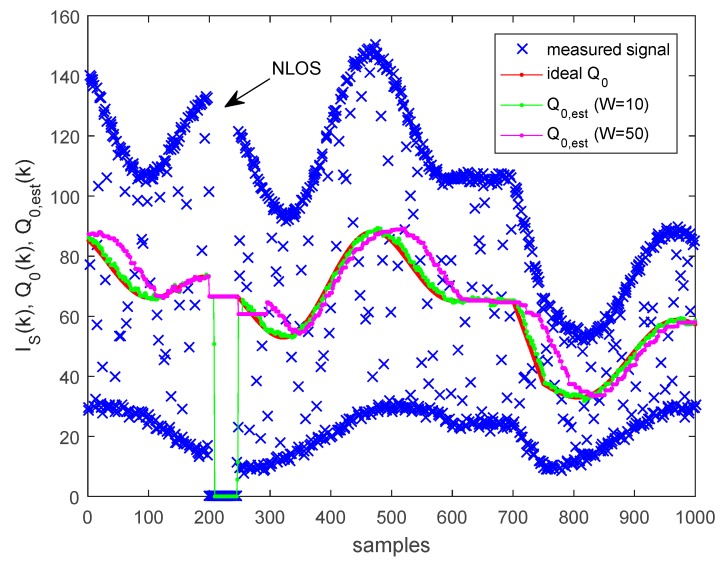
Adaptive estimation of threshold $Q_{0}$.

The operation of the adaptive estimator is illustrated in Figure 12. The varying input signal intensity, denoted by blue crosses, is synthetized with B=8 bits and camera aperture time of S=500 μs. The ideal $Q_{0}$ value is calculated using (8), and plotted in red. The signal contains additive noise (with standard deviation of 1 LSB). In the signal, there is a section where the line of sight is blocked (between samples 200 and 240), and a section where the amplitude is constant (between samples 600 and 700). In the experiment, two windows were used with W=10 and W=50.

Both estimators estimate the constant region well. The smaller window (shown in green) allows faster adaptation when the signal changes, thus the estimated threshold follows closely the ideal threshold. The larger window (shown in magenta) produces slower adaptation, and the delay between the ideal and estimated values is apparent.

When a non-line-of-sight (NLOS) situation occurs, the estimated threshold decreases to $A_{H}/2$ within a few samples (visible for both windows), and falls to zero not later than W samples after the start of the NLOS (visible for W=10). In both cases the estimate quickly recovers when the line of sight is restored. 

According to the experiment, the smaller window size provides faster and more accurate estimate; thus, in practice, a good choice is a window with size slightly above the minimum vale, e.g., around 10.

Notice that the adaptive mechanism only sets the threshold value and thus has no effect on the latency of the protocol.

### 4.4. Design Examples

To illustrate the practical usability of the theoretical results, two simple design examples will be presented.

#### 4.4.1. Example 1

*Input parameters:*

- Receiver: Let us use a camera with sampling frequency of 30 Hz. The exposure time is 1/4000 s. The jitter of the camera is smaller than 10 μs. The camera has 8-bit resolution. The maximum amplitude of the received signal changes between 80 and 120 LSB (for switched on LED). For the switched off LED, the received amplitude is approximately zero. The camera noise is 2 LSB.
- Transmitter: The software, which generates the transmitted signal, produces jitter smaller than 2 μs. The transmitted packet should contain a 16-bit value.

*Output parameter:*

- The optimal value of $T_{B}$ is to be determined for use in the generator software. We are interested in two cases: With and without adaptive thresholding.

*Design:*

First, let us approximate λ for the non-adaptive case. In this case, according to (8), the threshold is set to Q=50. In the calculation, the mean of $A_{H}=100\;\mathrm{LSB}$ will be used. Due to the amplitude variation, the ideal threshold varies between $40<Q_{0}<60$, thus ΔQ=−10…+10. Using the worst-case value of 10, according to (9):

(46) $$\lambda_{Q}≅2\frac{10\;\mathrm{LSB}}{100\;\mathrm{LSB}}\cdot 250\;\mu s=50\;\mu s$$

According to (10), the noise contribution is

(47) $$\lambda_{n}≅2\frac{2\;\mathrm{LSB}}{100\;\mathrm{LSB}}250\;\mu s=10\;\mu s$$

Using (11), the effect of the cumulative camera and transmitter jitter is:

(48) $$\lambda_{j}=2\left(10\;\mu s+2\;\mu s\right)=24\;\mu s$$

Thus, in the worst case, according to (20), with $\lambda_{f}=0$, the width of the unsafe interval is

(49) λ=50 μs+10 μs+24 μs=84 μs

The packet, using (27), contains M=4+2×16=36 symbols. Thus, according to (28), $T_{B}$ is approximately

(50) $$T_{B}≅\frac{T_{CAM}}{M}=\frac{1}{30\times 36}\;s≅1\;\mathrm{ms}$$

and, according to (38)

(51) $$a≅\frac{84\;\mu s}{1\;\mathrm{ms}}≅0.08<\frac{1}{7},$$

thus, according to (40), there exists a solution. Using (39), for design parameter c, the following constraint is given:

(52) 0.54<c<0.58.

In this case, the center of the interval is

(53) $$c_{opt}=0.56.$$

Using (53) for c, the exact value of $T_{B}$, calculated from (28), is the following:

(54) $$T_{B}=\frac{1}{30\times 36.56}\;s=911.7\;\mu s$$

which, according to (52), can vary in the following interval:

(55) $$911.2\;\mu s<T_{B}<912.2\;\mu s.$$

For the adaptive thresholding case, we assume that the rate of amplitude change is slow, thus we apply the estimate of ΔQ=1, leading to

(56) $$\lambda_{Q}≅2\frac{1\;\mathrm{LSB}}{100\;\mathrm{LSB}}\cdot 250\;\mu s=5\;\mu s$$

and

(57) λ=5 μs+10 μs+24 μs=37 μs.

The solution exists, since a≅0.04<1/7, with the following possible range for c:

(58) 0.52<c<0.59,

Resulting in the following range for $T_{B}$:

(59) $$910.9\;\mu s<T_{B}<912.8\;\mu s,$$

If no adaptive thresholding is used, according to (55), the clock can have ±0.5 μs error in each symbol, requiring relative frequency stability of $0.5\;\mu s/911.7\;\mu s≅5.5\times 10^{-4}$. In the adaptive case, the situation is slightly better, since, according to (59), the allowed error is ±0.9 μs error per symbol, i.e., relative frequency stability of $10^{-3}$. Since these values are technically not challenging, both solutions are robust in real applications.

#### 4.4.2. Example 2

1. *Input parameters:*

The camera and transmitter are the same as in Example 1. However, now the light intensity can change in a wide interval so that $A_{L}\approx 0$ and $A_{H}>128$ (saturation may occur). Let us determine the exposure time so that the performance of the protocol is guaranteed for any reasonable threshold value below $\min\left(A_{H},\;A_{max}\right)$, and any overload factor q>0.5. 

2. *Design:*

The unsafe interval, due to incorrect thresholding, is given by (18). The worst-case value of $\lambda_{Q}^{*}$, when q→∞, is the following:

(60) $$\lambda_{Q}^{*}≅\frac{2A_{H}^{*}}{A_{H}^{*}}S=2S.$$

The noise effect is characterized by (19), providing the worst-case value for the smallest possible amplitude (the saturation-free case):

(61) $$\lambda_{n}^{*}=\frac{2\;\mathrm{LSB}}{128\;\mathrm{LSB}}S.$$

Since $\lambda_{n}^{*}\ll \lambda_{Q}^{*}$, the effect of noise can be neglected. The effect of jitter is shown in (48). Thus, in the worst case, the width of the unsafe interval is

(62) $$\lambda =\lambda_{Q}^{*}+\lambda_{j}=2S+24\;\mu s.$$

The robust solution exists if (40) is true. Thus, using (38), the condition of the robust operation is the following:

(63) $$\lambda <\frac{1}{30\times 36\times 7}\;\mathrm{ms}\approx 130\;\mu s.$$

Using (62) and (63), the constraint for the aperture time is the following:

(64) $$S<\frac{130-24}{2}\;\mu s\approx 50\;\mu s.$$

## 5. Evaluation

In this section, the performance of the proposed RUPSOOK protocol is evaluated using simulations and real measurements.

For evaluation purposes, instead of c, a more practical parameter, called samples per bit (SPB), will be used. Since the length of one bit is $2T_{B},$ the number of samples per bit is, using (29), can be expressed as follows:

(65) $$SPB=\frac{2T_{B}}{\delta }=\frac{2}{c}.$$

Notice that according to (42), and the examples in Section 4.4, the ideal value of c is around 0.55…0.56, which corresponds with ideal samples per bit value of SPB≅3.6.

Table 2 shows the main technical parameters, used in the simulations and measurements.

### 5.1. Simulations

The behavior of the proposed protocol was analyzed in a well-controlled simulation environment. The sampled signal was simulated using an ideal square-wave blinking signal and a camera model according to (7). The resulting signal was similar to the trapezoid signal shown in Figure 4a, with $T_{B}=T_{DATA}/2$, with tunable parameters $T_{B}$ and S. By varying the sampling rate, various SPB values were set, and the variation of the sampling frequency allowed the simulation of jitter and frequency error as well. With additive noise, the effect of noise was modelled.

The camera frequency was set to 30 Hz, the simulated aperture time was 100 μs, and the received signal amplitude was set between 40 and 160. The threshold Q was varied from 0 to 255, and the transmitter frequency was tuned to provide samples per bit (SPB) between 2.5 and 5. In each simulation, the same transmission sequence with 200 packets was used and the packet error rate (PER) was calculated as follows:

(66) $$PER=\frac{number\;of\;bad\;packets}{number\;of\;all\;packets}.$$

**Figure 13:**
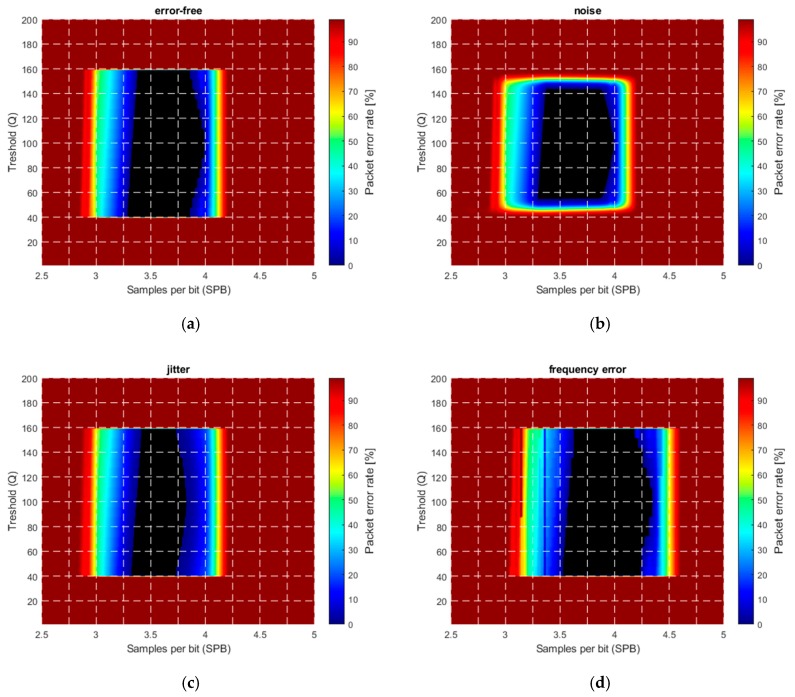

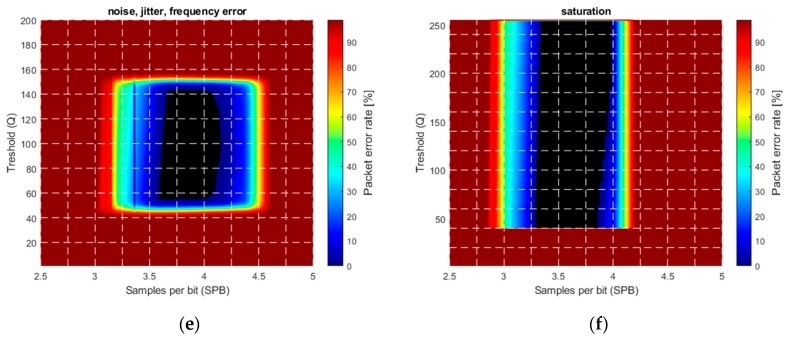
Simulated packet error rate (PER) in the presence of various error sources. (**a**) Ideal case, (**b**) noise with σ=5 LSB, (**c**) ±100 μs jitter, (**d**) camera frequency error of with $\Delta f_{CAM}=-0.1\;\mathrm{Hz}$, (**e**) combined noise, jitter, and frequency error, (**f**) saturation.

In Figure 13, the PER is shown as a function of the threshold Q and samples per bit SPB. Figure 13a shows the ideal case, where no error source is present. The central part, shown by black, is error free (PER=0). The error-free region is located around SPB=3.6, corresponding well with the theoretical results. As it was expected, the protocol operates well with thresholds between the minimum and maximum. 

Figure 13b shows the effect of additive noise with variance σ=5 LSB. The error-free region decreased by approximately 3σ LSB around the threshold values corresponding to the minimum and maximum signal amplitude (i.e., the top and bottom of the error-free region).

For better visibility, in the simulations, a high jitter value in the range of ±100 μs, was used, with uniform distribution. The effect is shown in Figure 13c: The shrinking of the error-free region on both the left-hand side and right-hand side is clearly observable.

The frequency error was modelled as a constant bias in the camera frequency: The camera frequency was set to 29.9 Hz. The effect is visible in Figure 13d, where the original error-free region is shifted to the right. The explanation is the following: RUPSOOK uses a constant frequency difference between the camera and the transmitter, according to (28) and (29). This frequency difference is modified by the constant frequency error of the camera, thus the designed SPB value is different from the nominal value, thus the error-free region is shifted. If the sampling of camera is faster/slower than expected, then the real SPB is higher/lower than the nominal, causing shift of the error-free region to the left/right. The theoretical relationship between the nominal *SPB*, the real SPB’, and the frequency error Δf is the following:

(67) $$SPB^{\prime }≅\frac{SPB}{1+SPB\frac{1}{2T_{B}}\frac{\Delta f_{cam}}{f_{cam}^{2}}}≅SPB-SPB^{2}\frac{1}{2T_{B}}\frac{\Delta f_{cam}}{f_{cam}^{2}}.$$

As an example, if the nominal SPB is 3.5 and the frequency error is 0.1 Hz, then the real ${SPB}^{\prime }$ is 3.2, thus the values corresponding to SPB=3.5 in Figure 13d correspond to values SPB=3.2 in Figure 13a, producing a visible shift. Notice that, according to (67), the value of the shift depends on the SPB: The larger the SPB, the larger the shift.

The effect of multiple error sources, including noise, jitter, and frequency error is shown in Figure 13e. The levels of the disturbances are equal to those of the individual sources presented in Figure 13a–d. The combined effect, containing shrinking and shifting of the error-free region, is obvious.

The effect of saturation is illustrated in Figure 13f. In the example, the signal values changed between 40 and 400, saturated at 255. Without saturation (up to amplitude 400), the figure would be an enlarged version of the saturation-free case of Figure 13a, but due to saturation, it is truncated at the saturation level of 255. Thus, Figure 13f is the stretched version of the lower half of Figure 13a, in agreement with the results of Section 3.2.5.

### 5.2. Measurement Setup

**Figure 14:**
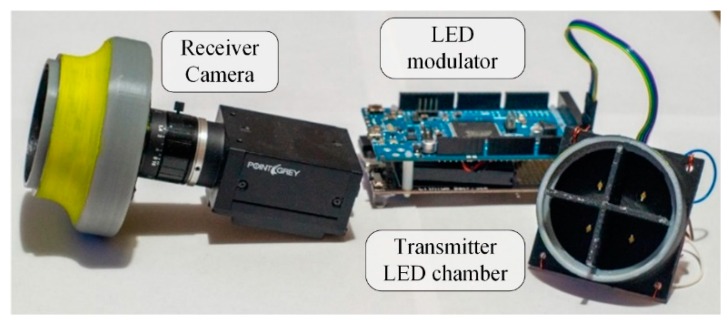
The setup for laboratory measurements.

**Figure 15:**
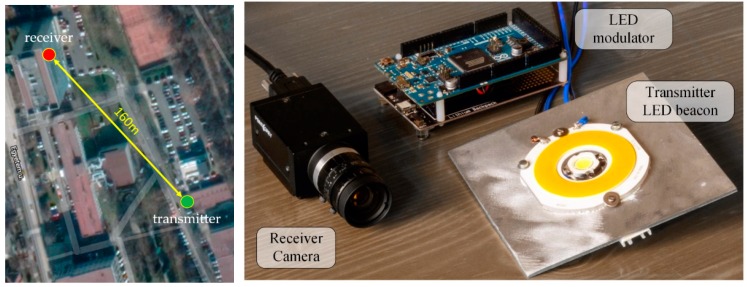
Long-distance measurement setup and a photo of the equipment.

During the tests, two measurement setups were utilized: A well-controlled laboratory environment and a long-distance outdoor setup. In the laboratory setup, the camera and the low-power transmitters were built into an enclosure blocking external light, where the minimum and maximum light intensity, along with the noise level, can be controlled. The photo of the equipment is shown in Figure 14. In the long-distance setup, power LEDs were used, as transmitters. The map of the premises and the photo of the equipment are shown in Figure 15.

The main technical parameters of the measurements are shown in Table 2. Notice that in Table 2, the value of $T_{CAM}$ is the nominal value, but the camera provided slightly different sampling period; thus, parameter $T_{B}$ was tuned while measuring the actual value of parameter SPB. The measurement method for SPB is described in Section 5.3.

### 5.3. Measurement of the SPB Value

**Figure 16:**
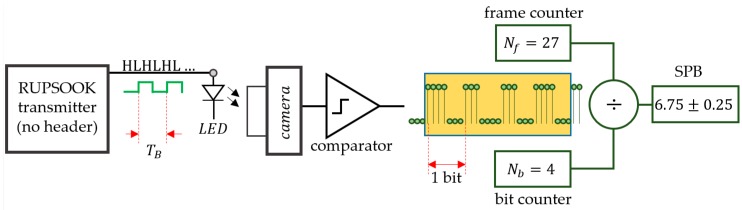
Measurement setup to determine the SPB value.

For the tests, the exact SPB value need to be determined. Parameter $T_{B}$ of the transmitter can be measured easily with high precision, using a time/frequency meter, but for most cameras, the measurement of the exact sampling frequency is troublesome. Thus, the value of δ is difficult to obtain. Instead, a measurement setup was used to directly measure the SPB, as shown in Figure 16.

The transmitter is the RUPSOOK transmitter under test, but during the SPB measurement, the packets do not contain header and the same symbols (e.g., MARK symbols) are transmitted, resulting in a continuous alternating sequence of HLHLHL. The received samples are binarized by a comparator. The measurement is performed using two counters: One counter contains the number of received bits $N_{b}$, while the other counts the number of received frames (or samples) $N_{f}$. After each received full bit, the ratio of the samples and bits is calculated, providing an estimate for the SPB value:

(68) $$SPB=\frac{N_{f}}{N_{b}}\pm \frac{1}{N_{b}}.$$

Figure 16 shows the result after the reception of 4 full bits, containing 27 samples. Note that the counters should be cleared when the transmitter frequency is changed. Using the real-time feedback of SPB values, the required SPB can be easily set by tuning the transmitter’s clock frequency.

Notice that the speed of measurement depends on the actual SPB value and the required precision. For example, for SPB=3, each bit contains three samples, thus samples corresponding to one bit are collected in 0.1 s, using $f_{S}=30\;\mathrm{Hz}$. In order to provide measurement accuracy of ±0.05, according to (66), $N_{b}=20$ is necessary, thus the measurement time is 20×0.1 s=2 s.

### 5.4. Laboratory Measurements

The laboratory measurements contained two setups, using aperture times of 100 μs and 15 μs. The technical parameters are listed for both setups in Table 2. In each test different *SPB* values were set, ranging from 2.75 to 4.25. The packet error rate (PER), as a function of parameters SPB and Q, was calculated.

**Figure 17:**
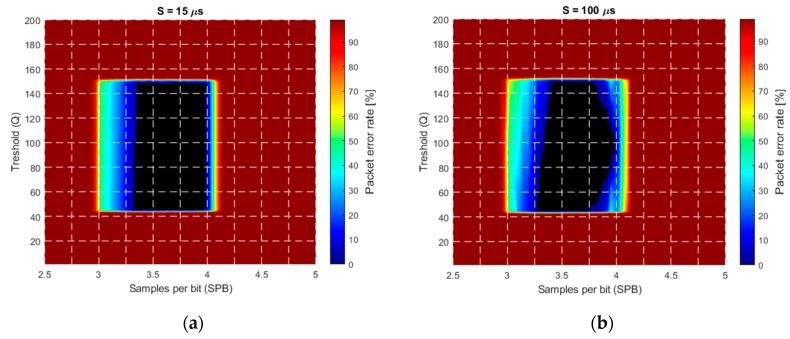
The packet error rate as a function of parameters SPB and Q. (**a**) S=15 μs, (**b**) S=100 μs.

Figure 17 shows the PER as a function of SPB and Q, for both setups. The shape and size of the error-free regions resemble to those of the simulation results. In case of smaller aperture time, the tilting on the left- and right-hand side of the error-free region is narrower. The robust operation is apparent: The PER is zero for a wide range of parameters SPB and Q, as shown by the black area in the center.

Notice that the robust operation region provides guaranteed response time. For example, in the case of SPB=3.5, the decoding of 11-bit long packet requires 39 samples. Since, in the worst case, the reception begins slightly after the start of the first header symbol, almost two packets must be received for the first detection. In this case, considering the utilized camera frequency, the worst-case guaranteed response time will be 2.6 s.

### 5.5. Long-Distance Measurement

**Figure 18:**
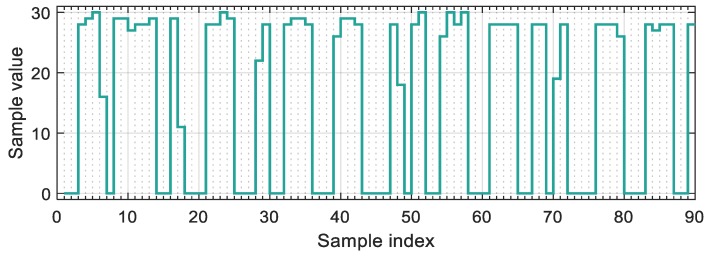
Example samples from the long-distance measurements.

A long-distance measurement was also conducted to check the robustness of RUPSOOK in extreme conditions. The camera was placed in a ninth-floor window, while the transmitter was deployed at the side of a nearby road (see Figure 15). The distance between the transmitter and the camera was 160 m. In this experiment, no additional noise was added to the measurement. The noise was observable only at state H (with standard deviation of 1.7 LSB), state L was always zero. A sample packet can be seen in Figure 18. Notice that most of the samples are around zero and 30 but there are numerous samples in between: These were taken in unsafe intervals.

**Figure 19:**
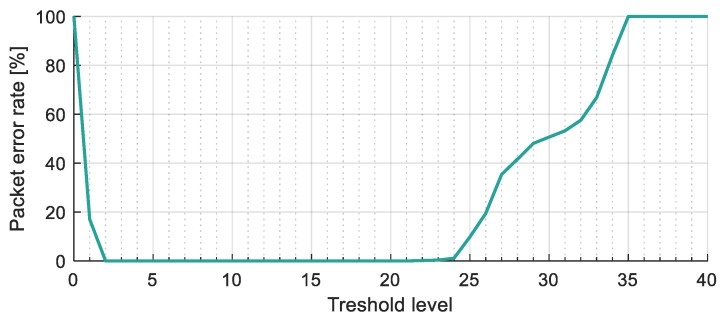
The packet error rate of the long-distance measurement, as a function of parameter Q.

The PER is shown in Figure 19, as a function of parameter Q. The performance of the protocol was perfect (zero error) with parameters 1≤Q≤20, for the approximately 38,000 transmitted bits.

### 5.6. Comparison

The main performance properties of UFSOOK [8], UPSOOK [9], the undersampled 64-PAM [10], and the proposed RUPSOOK protocol are listed in Table 3. The highest spectral efficiency belongs to U-64-PAM, where the PAM coding allows the coding of multiple (8) bits into one symbol. In this respect, RUPSOOK has the lowest performance with only 0.28 bits/sample. The covered distance is short in the case of U-64-PAM, due to the sensitive coding. Both UFSOOK and UPSOOK performs better, due to the OOK coding, but the observed sensitivity of the header to distance reduces the possible distance coverage [9]. RUPSOOK’s better performance is due to its header structure, where there is no need for a different symbol. The theoretical PER is zero for RUPSOOK, with correct settings. The range of correct settings is wide; thus, the protocol is robust. The only correct setting for the other protocols is the tight synchronization, which is not practical. Thus, RUPSOOK is able to guarantee quality of service (QoS) for the response time, while the other protocols are not. The worst-case response time is constant for RUPSOOK, while this time is inversely proportional with the frequency error δ for other protocols.

## 6. Conclusions

A potential drawback of undersampled protocols was highlighted and discussed. It was demonstrated that in the non-synchronized case, small-frequency differences between the clocks of the transmitter and receiver cause periodic failures in the detection. The properties of the failure were analyzed as a function of protocol parameters (aperture time, signal frequency, threshold in the decoder) and various error sources (noise, jitter, frequency slip).

A novel robust protocol, called RUPSOOK, was proposed, which guarantees correct decoding in the presence of physical error sources and a wide range of protocol parameters, without using any error correcting codes. The performance of the protocol was demonstrated by simulations and real measurements. The new protocol provides robust operation, guaranteed response time, and increased communication range, at a price of a somewhat decreased communication speed. The adaptive version of the protocol also provides robust operation when the amplitude of the signal is changing.

## Figures and Tables

**Table 1:** Theoretical results and measured performance properties of the UPSOOK protocol.

Q	Theoretical		Measured	
	$T_{P}$	$T_{unsafe}$	$T_{P}$	$T_{unsafe}$
20	694 s	33.5 s	729 s	30 s
50	694 s	8.5 s	718 s	7 s
58	694 s	1.8 s	721 s	1 s

**Table 2:** Technical parameters of the measurements.

Parameter	Simulations	Laboratory I.	Laboratory II.	Long Distance
$T_{CAM}\;\left(s\right)$	1/30	1/30	1/30	1/30
S (μs)	100	100	15	100
f-number	n.a.	f/8	f/2.8	f/2
focal length (mm)	n.a.	2.7	2.7	6
B (bits)	5 + 2 header	9 + 2 header	9 + 2 header	20 + 2 header
SPB (c)	2.5 … 5.0 (0.4 … 0.8)	2.75 … 4.25 (0.47 … 0.73)	2.75 … 4.75 (0.47 … 0.73)	3.65 (0.55)
$T_{B}\;\left(\mathrm{ms}\right)$	≅2.381	≅1.515	≅1.515	≅0.7575
Q (LSB)	0…255	0 … 255	0 … 255	0 … 40
$A_{L};\;A_{H}\;\left(\mathrm{LSB}\right)$	40; 160	42; 155	43; 154	0; 28–36 (varied)
$std_{noise}\;\left(\mathrm{LSB}\right)$	5 (controlled)	0.8 (L), 2.31 (H)	0.9 (L), 2.7 (H)	0 (L), 1.7 (H)
$P_{LED}\;\left(W\right)$	n.a.	0.0015	0.0015	4.9
total #bits sent	1000/experiment	1800/experiment	1800/experiment	38,280

**Table 3:** Performance properties of UFSOOK [8], UPSOOK [9], undersampled 64-PAM [10], and robust UPSOOK (RUPSOOK).

	UFSOOK	UPSOOK	UPAM-64	RUPSOOK
bits/sample	0.5	1	8	0.28
covered distance	medium	medium	low	high
theoretical PER	>0	>0	>0	=0
QoS	no	no	no	yes
Worst case response time	O(1/δ)	O(1/δ)	O(1/δ)	O(1)
